# Inhibition of PTEN Gene Expression by Oncogenic miR-23b-3p in Renal Cancer

**DOI:** 10.1371/journal.pone.0050203

**Published:** 2012-11-26

**Authors:** Mohd Saif Zaman, Sobha Thamminana, Varahram Shahryari, Takeshi Chiyomaru, Guoren Deng, Sharanjot Saini, Shahana Majid, Shinichiro Fukuhara, Inik Chang, Sumit Arora, Hiroshi Hirata, Koji Ueno, Kamaldeep Singh, Yuichiro Tanaka, Rajvir Dahiya

**Affiliations:** Department of Urology, San Francisco Veterans Affairs Medical Center and University of California San Francisco, San Francisco, California, United States of America; Wayne State University School of Medicine, United States of America

## Abstract

**Background:**

miR-23b is located on chromosome number 9 and plays different roles in different organs especially with regards to cancer development. However, the functional significance of miR-23b-3p in renal cell carcinoma (RCC) has not been reported.

**Methods and Results:**

We measured miR-23b-3p levels in 29 pairs of renal cell carcinoma and their normal matched tissues using real-time PCR. The expression level of miR-23b-3p was correlated with the 5 year survival rate of renal cancer patients. In 15 cases (52%), miR-23b-3p expression was found to be high. All patients with moderate to low miR-23b-3p expression survived 5 years, while those with high miR-23b-3p expression, only 50% survived. After knocking down miRNA-23b-3p expression in RCC cell lines, there was an induction of apoptosis and reduced invasive capabilities. MiR-23b-3p was shown to directly target PTEN gene through 3′UTR reporter assays. Inhibition of miR-23b-3p induces PTEN gene expression with a concomitant reduction in PI3-kinase, total Akt and IL-32. Immunohistochemistry showed the lack of PTEN protein expression in cancerous regions of tissue samples where the expression of miR-23b-3p was high. We studied the *in vitro* effects of the dietary chemo preventive agent genistein on miR-23b-3p expression and found that it inhibited expression of miR-23b-3p in RCC cell lines.

**Conclusions:**

The current study shows that miR-23b-3p is an oncogenic miRNA and inhibits PTEN tumor suppressor gene in RCC. Therefore, inhibition of miR-23b-3p may be a useful therapeutic target for the treatment of renal cell carcinoma.

## Introduction

miRNAs have been shown to be involved in various kidney diseases [1]. miR-23b is located on chromosome number 9 in a cluster with miR-27b and miR-24-1 [2]. It has been shown to play different roles in different organs especially with regards to cancer development. In cancers like bladder and oral squamous cell carcinoma [3]–[4] it has been shown to be over expressed. In renal cancer miR-23b-5p acts as a potential oncomir by suppressing proline oxidase, which is a novel tumor suppressor protein [5]. Whereas in prostate cancer [6], it plays the role of a tumor suppressor microRNA as it is targeted by the oncogenic transcription factor c-myc. This eventually results in an increase in the protein expression of the pro-cancerous enzyme mitochondrial glutaminase, which is a target for miR-23b [7]. In breast cancer it has been shown to be up regulated with miR-27b and miR-24-1 by the oncosuppressor transcription factor ERβ [8]. Similarly, in hepatocellular carcinoma it acts as a tumor suppressor by targeting the Urokinase-type plasminogen activator (uPA) and c-met resulting in decreased migration and proliferation of HCC cells [9]. In colon cancer it functions in suppressing cancer metastasis by targeting prometastatic genes FZD7 or MAP3k1 [10].

In this study, we examined the role of miR-23b-3p in renal clear cell carcinoma. We found that expression of miR-23b-3p was increased in A-498 and Caki-2 cell lines and a majority of tissue samples. Increased expression also correlated with lower survival rate for renal cell carcinoma patients. Inhibition of miR-23b-3p expression in A-498 and Caki-2 cells caused an increase in apoptosis and a decrease in invasive properties. PTEN protein expression was found to be increased after knocking down miR-23b-3p in A-498 cells with a concomitant decrease in the expression of PI3-kinase, total Akt and IL-32. The PTEN gene was shown to be a direct target of miR-23b-3p by 3′ luciferase reporter assays. Immunohistochemistry showed the lack of PTEN protein expression in cancerous regions of tissue samples where the expression of miR-23b-3p was high.

## Results

### miR-23b-3p is Up Regulated in Renal Carcinoma Tissue Samples and Renal Cancer Cell Lines

**Figure 1:**
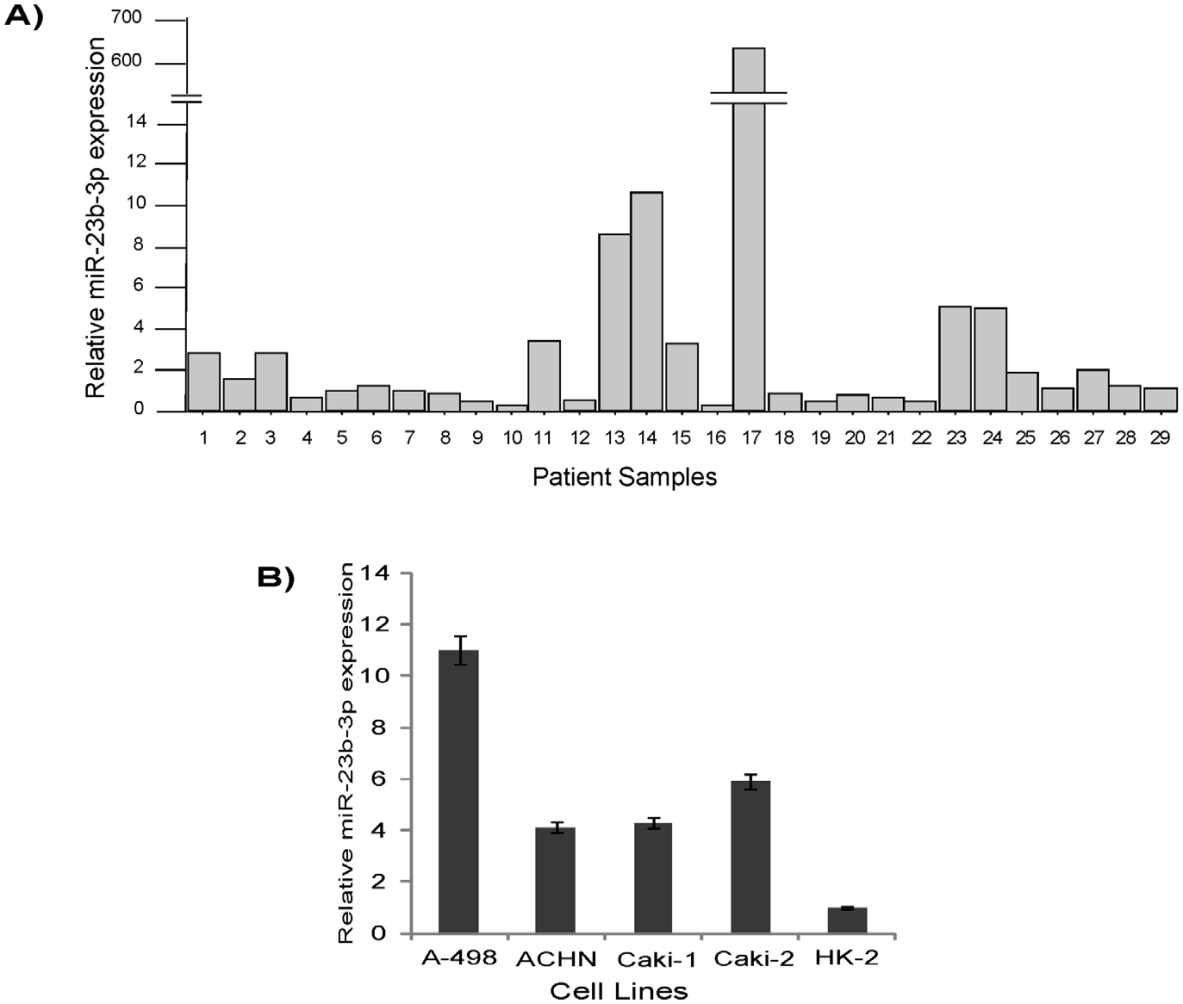
qRT-PCR expression of miR-23b-3p in renal cancer tissues and cell lines. A) Ratio of T/N expression in tissue samples was found to be high in a majority of samples. B) A-498 and Caki-2 cells had high expression of miR-23b-3p as compared to non malignant normal HK-2 cells (T-Tumor,N-Normal), [p value (A) 0.032; p value (B) 0.018)].

To understand the clinical relevance of miR-23b in kidney cancer we measured miR-23b-3p levels in 29 pairs of human kidney cancers (all clear cell renal cell carcinoma) and matched normal tissues by real-time PCR. In 15 cases (52%), miR-23b-3p was found to be increased (T/N [Tumor/Normal] was greater than 1.2). In 5 samples (17%) the expression was moderate (T/N 0.8–1.2). Expression of miR-23b-3p was also measured in cultured renal cancer cell lines, A-498, ACHN, Caki-1, Caki-2 and non malignant normal HK-2 cells. miR-23b-3p expression in A-498 cells was approximately 11x that of HK-2 cells while that of ACHN was 4.1x, Caki-1 4.8x, and Caki-2 5.8x (Figure 1).

### Correlation of miR-23b-3p Expression with Survival in Renal Cancer

**Figure 2:**
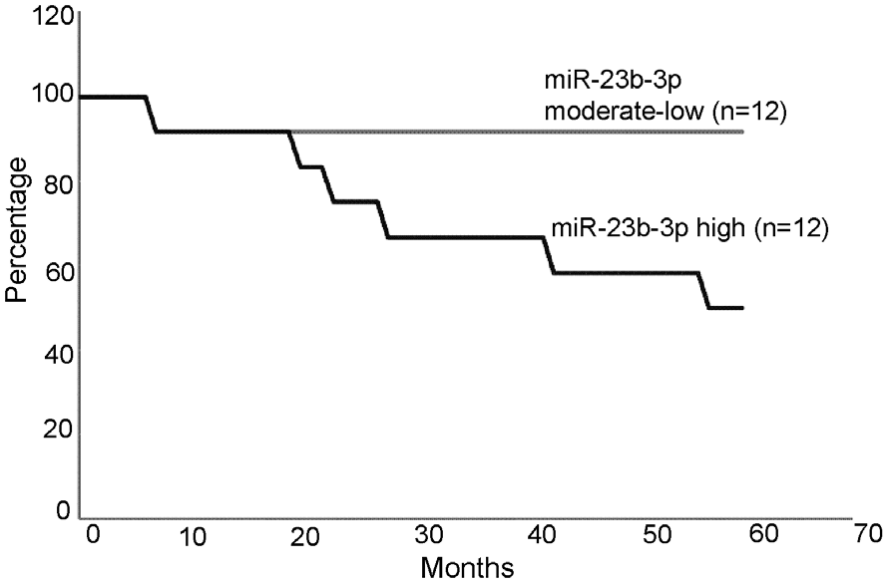
Correlation of miR-23b-3p expression with 5 year survival of renal cell carcinoma patients (p value 0.017).

The expression level of miR-23b-3p correlated with 5 year survival of the patients. For those patients with moderate to low miR-23b-3p expression (n = 12) (Figure 2), all survived 5 years after surgery, while those with high miR-23b-3p (n = 12) expression, only 50% survived (Figure 2). The survival curve was based on the number of patients (24) for which we had survival information out of a total of 29 patients. No correlation between tumor grade, stage and miRNA-23b-3p levels were observed.

### Functional Role of miR-23b-3p in A-498 Cells

**Figure 3:**
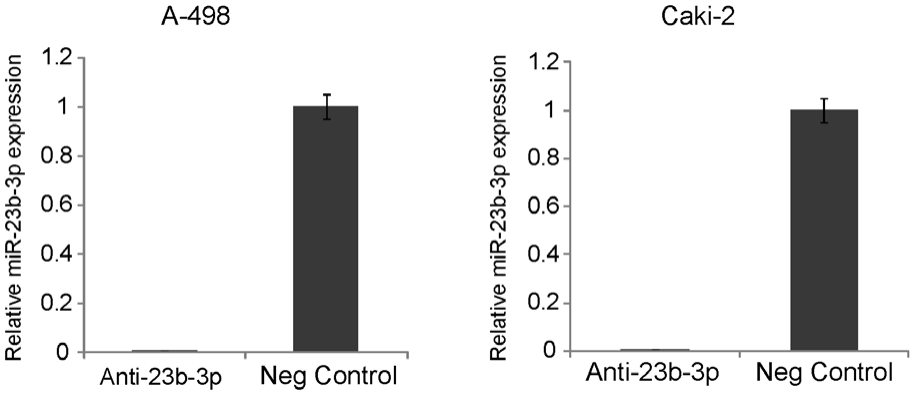
Expression of miR-23b-3p after knock down. Expression of miR-23b-3p was decreased by more than 99% after its inhibition in both A-498 (A) and Caki-2 cells (B).

To elucidate the functional role of miR-23b-3p in renal cancer we knocked down the expression of miR-23b-3p in A-498 and Caki-2 renal cancer cells with a commercially available miR-23b-3p inhibitor. Anti-miR-Negative control #1 was also used for these experiments. The miR-23b-3p level was reduced by more than 99%, as compared to the negative control in A-498 cells (Figure 3a). Anti-miR-23b-3p transfection into Caki-2 cells also reduced the level of miR-23b-3p expression in these cells (Figure 3b).

### Effect on Cell Cycle and Apoptosis

**Figure 4:**
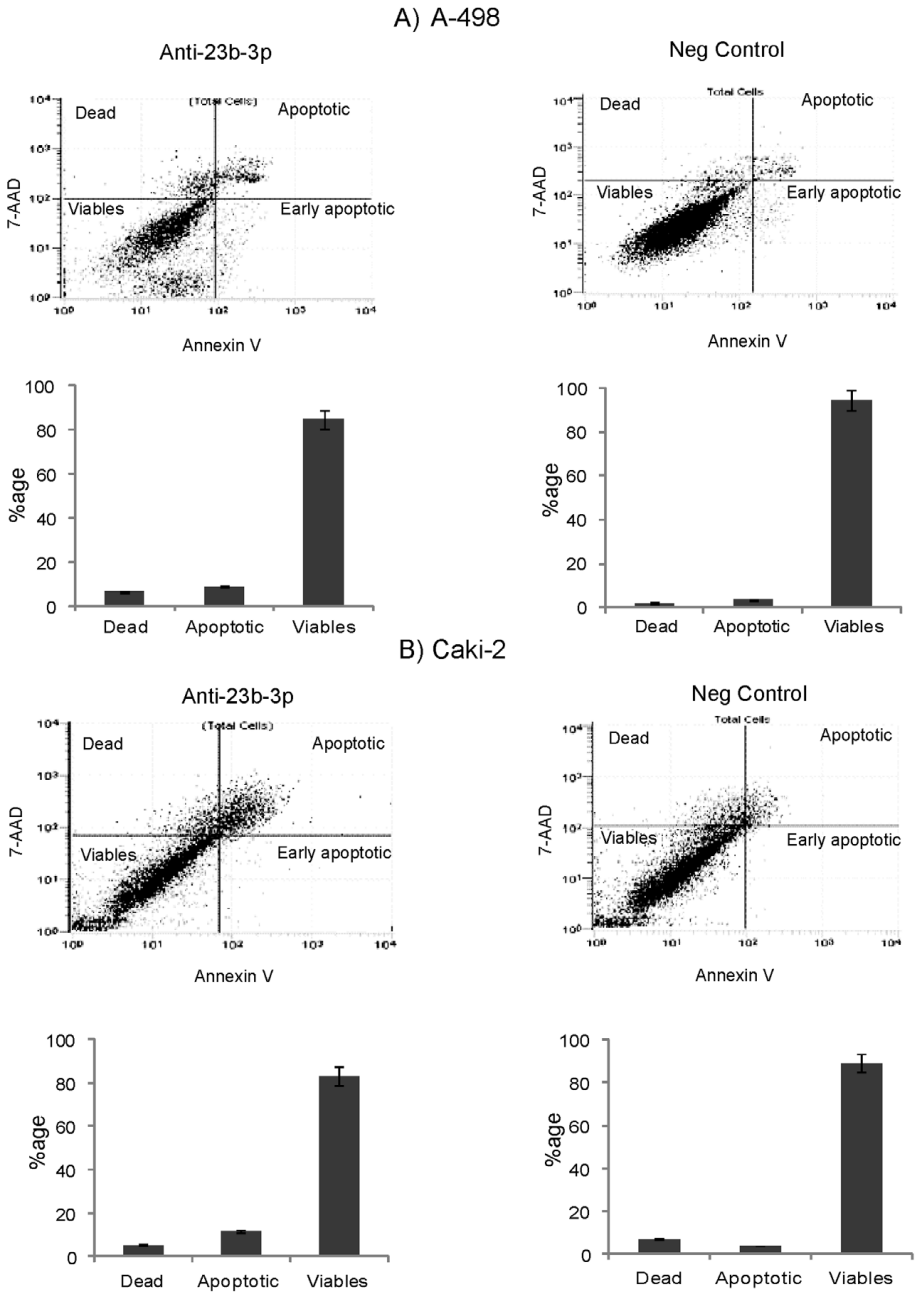
Effect on apoptosis after miR-23b-3p knock down in A-498 and Caki-2cells. A) Apoptosis assay showed a significant increase in the number of total apoptotic cells (8.87%) in the anti- miR-23b-3p inhibitor transfected cells as compared to the negative control (3.37%) in A-498 cells (p value 0.029). B) In Caki-2 cells the total number of apoptotic cells (11.74%) in the anti- miR-23b-3p inhibitor transfected cells as compared to the negative control (3.68%) (p value 0.030).

The effects of miR-23b-3p knock down on the cell cycle and apoptosis were analyzed by flow cytometry. Apoptosis assay showed a significant increase in the number of total apoptotic cells (early apoptotic plus apoptotic) in both A-498 (8.87%) (Figure 4a) and Caki-2 cells (11.74%) (Figure 4b) with miR-23b-3p knock down as compared to the negative control [A-498 (3.37%) and Caki-2 (3.68%)]. There were no significant changes in the cell cycle for both A-498 and Caki-2 cells (data not shown).

### Effect on Cell Invasion and Migration Properties

**Figure 5:**
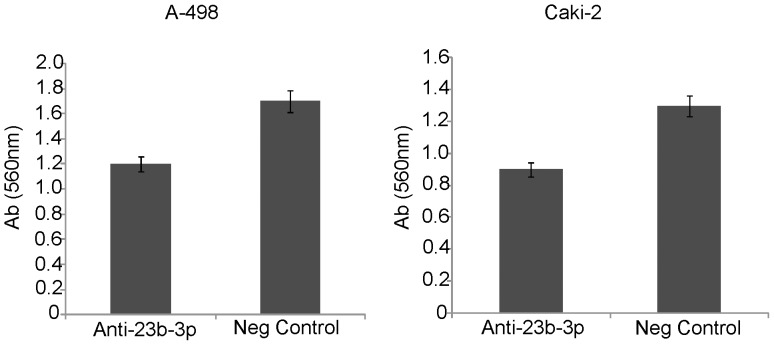
Effect of miR-23b-3p knock down on cell invasion in A-498 and Caki-2 cells. Invasive properties of A-498 (A) and Caki-2 (B) cells were decreased by 30% after miR-23b-3p knock down compared to controls. Y-axis-Absorbance at 560 nm [p value(A) 0.026; p value(B) 0.030].

To determine whether miR-23b-3p affects renal cancer cell migration and invasion a cytoselect 24-well cell migration and invasion kit was used. A-498 and Caki-2 cells were transfected with anti-miR-23b-3p inhibitor and anti-miR-Negative control. Both A-498 and Caki-2 cells showed a 30% decrease in cell invasion in the samples where miR-23b-3p was inhibited as compared to negative controls (Figure 5a and 5b). No significant change in migration was observed in both A-498 and Caki-2 cells (data not shown).

### PTEN is Increased After Knock Down of miR-23b-3p in A-498 Cells

**Figure 6:**
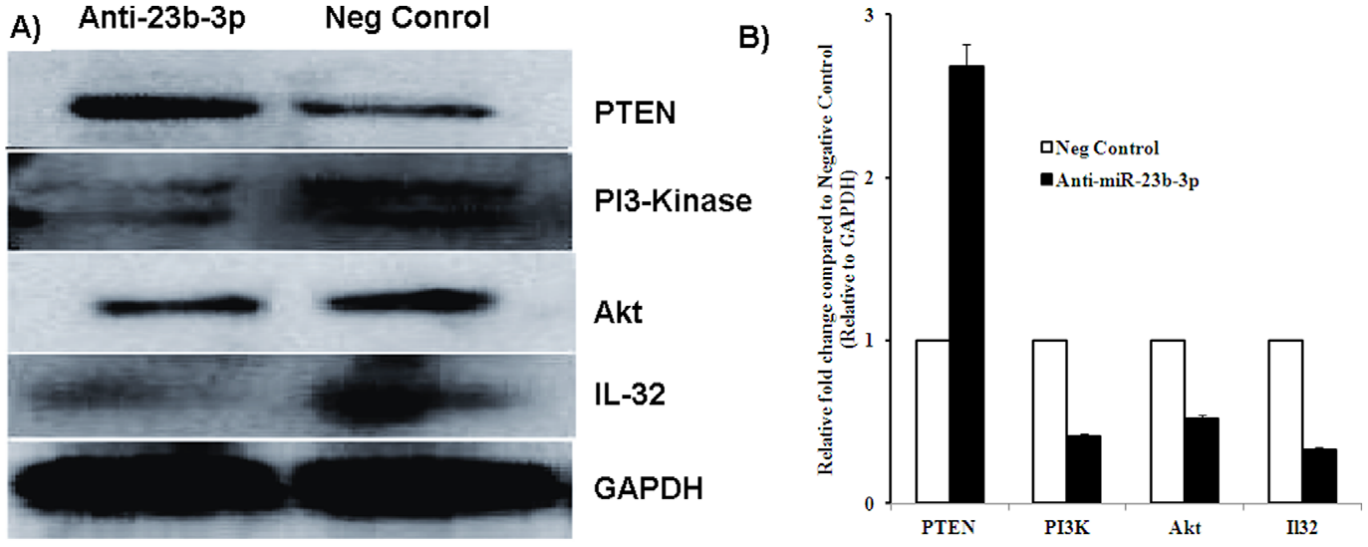
Western analysis of apoptosis and invasion genes after knock down of miR-23b-3p in A-498 cells. A) Expression of PTEN, PI3-kinase, Akt and IL-32 in A-498 cells, compared to negative control. GAPDH was used as a normalizing control. B) Quantitative data for Western blot analysis. PTEN was up regulated, whereas PI3-kinase, Akt and IL-32 expression were down regulated.

To see the effect of miR-23b-3p expression on different genes involved in apoptosis and invasion we checked the protein expression of several genes involved in these processes. The pro apoptotic gene PTEN was found to be up regulated after miR-23b-3p knock down in A-498 cells (Figure 6A). We also found a decrease in the protein levels of PI3-kinase and total Akt. Interestingly, a decrease in the expression of the pro inflammatory cytokine IL32 was also observed after miR-23b-3p knock down. The protein expression levels were quantified by optical densitometry using ImageJ Software version 1.36b (http://rsb.info.nih.gov/ij/). Fold change was calculated as the ratio between the net intensity of each sample transfected with anti-miR-23b-3p divided by GAPDH and the negative control transfected samples divided by the GAPDH (Anti-miR-23b-3p transfected samples/GAPDH)/(Neg Control miR transfected samples/GAPDH) (Figure 6B).

### PTEN is a Direct Target of miR-23b-3p

**Figure 7:**
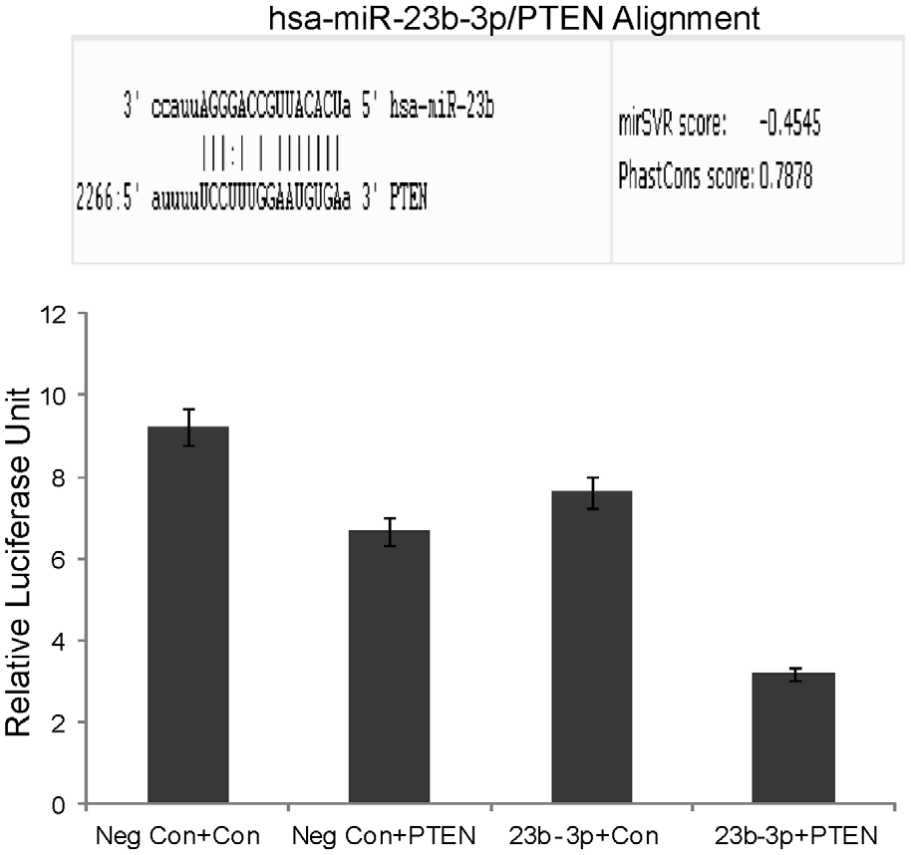
PTEN is a direct target of miR-23b-3p. A) Software analysis shows sequences complementary to the seed sequences of miR-23b-3p in the 3′ UTR of PTEN gene. B) Luciferase assay showed a decrease in relative luciferase units in samples co transfected with miR-23b-3p was and PTEN 3′ UTR gene construct as compared to control constructs (Neg Con-negative control; Con-Control plasmid construct lacking PTEN 3′UTR sites for miR-23b-3p; PTEN-PTEN plasmid construct having PTEN gene 3′UTR sites for miR-23b-3p; (p value 0.017).

Since a significant increase in apoptosis was observed in both A-498 and Caki-2 cells and increased PTEN expression in A-498 cells after miR-23b-3p knock down, we looked to see if miR-23b-3p targets the pro apoptotic gene PTEN. Using TargetScan (targetscan.org) and microrna.org we identified complementary sequences to miR-23b in the 3′UTR of the PTEN gene (Figure 7a). We carried out luciferase reporter assays using a 3′PTEN construct with miR-23b-3p or miR-Control- expressing A-498 cells and observed a consistent reduction of luciferase activity (Figure 7b) in miR-23b-3p transfectants suggesting that miR-23b-3p represses PTEN directly.

### Expression of PTEN Protein in Normal and Cancer Regions of Tissue Samples having a High Expression of miR-23b-3p

**Figure 8:**
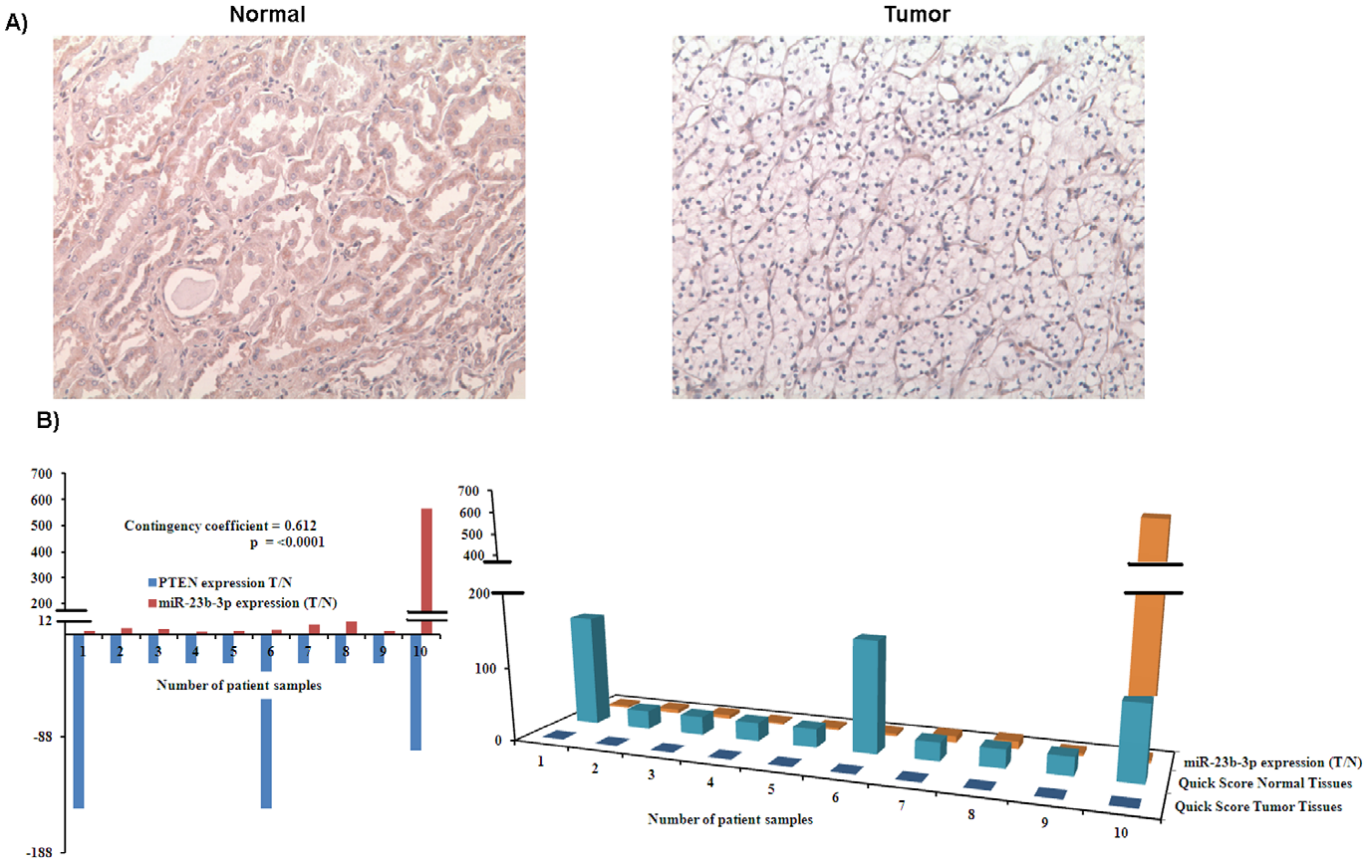
Expression of PTEN protein in normal and cancer regions of tissue samples having high expression of miR-23b-3p. A) Representative pictures of Immunohistochemistry (IHC) analyses. B) Graphical representation of the data showing that PTEN expression was high in normal regions of renal cancer patient samples whereas in cancerous regions having high miR-23b-3p expression PTEN was not observed.

To further confirm that PTEN is a target of miR-23b-3p we checked for expression of PTEN protein in normal and cancer regions of patient tissue samples which had a high expression of miR-23b-3p, using immunohistochemistry (IHC). Ten samples having high miR-23b-3p expression were selected. A representative example of PTEN immunohistochemistry is shown in Figure 8A.

IHC staining results for tissues were graded according to quick score (percent cells stained × intensity of stain). Quick score obtained from the tumor samples was normalized with the quick score of their normal samples (T/N) and Chi-square test was performed with the miR-23b-3p expression levels in the same patient samples (Figure 8B). All normal samples expressed PTEN protein though at varied levels. PTEN staining was absent from the tumor samples that expressed higher levels of miR-23b-3p (p<0.001) (Figure 8B).

### Genistein Decreases the Expression of miR-23b-3p in A-498 Cells

**Figure 9:**
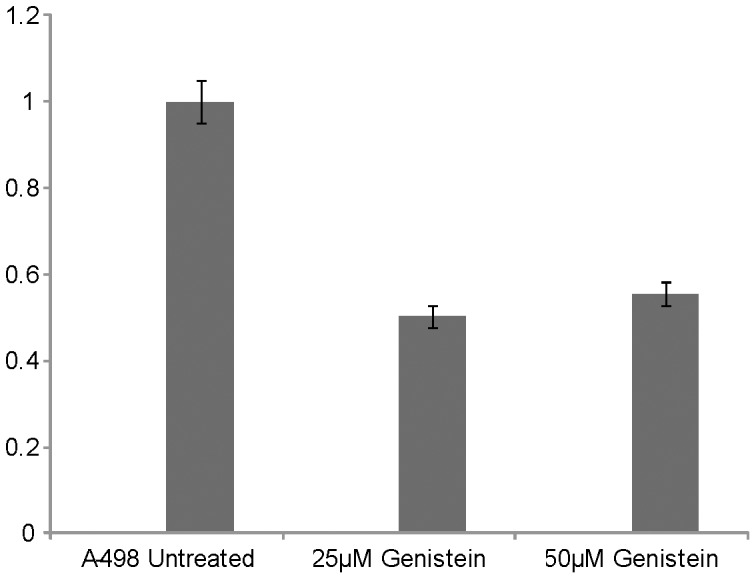
*In vitro* effect of genistein on the expression of miR-23b-3p. miR-23b-3p expression was reduced by 50% in 25 µM genistein treated A-498 cells as compared to untreated cells, 50 µM genistein decreased miR-23b-3p expression by 45% (p value 0.030).

We also examined the effect of the chemoprevention agent, genistein (a soy product), on the expression of miR-23b-3p in A-498 cells. Treatment with genistein (25 µM) for 96 hours decreased the expression of miR-23b-3p by 50%, whereas a higher concentration of genistein (50 µM) for the same period of time decreased expression by 45% (Figure 9).

## Discussion

The present study shows that miR-23b-3p functions as an oncogenic microRNA in renal cell carcinoma. Its expression is increased in renal cell carcinoma cell lines and tissue samples (clear cell carcinoma) as compared to normal renal cells and matched normal tissues, respectively. In addition we found that high levels of miR-23b-3p lead to lower survival for renal cancer patients. To study the functional role of miR-23b-3p its expression was knocked down in A-498 and Caki-2 renal cancer cell lines. This led to an increase in the total number of cells undergoing apoptosis in both cell lines compared to controls. However, there was no significant change in the cell cycle. In addition A-498 and Caki-2 cells showed decreased cell invasion in miR-23b-3p knocked down cells but no significant change in migration. Western analysis of miR-23b-3p knock down showed increased PTEN expression and concomitant decreases in the expression of PI3-kinase, Akt and IL-32. PTEN was found to be a direct target of miR-23b-3p through reporter gene assays.

The significant change in the number of apoptotic cells after knock down of miR-23b-3p in both A-498 and Caki-2 cells lead us to search for genes and pathways involved in apoptosis. Previous studies have shown that PTEN can induce apoptosis through a PI3-kinase dependent pathway [11]. PTEN acts as a tumor suppressor gene through the action of its phosphatase protein product [12], degrading PIP3 to inactive phosphatidylinositol (4,5)-diphosphate PIP2 [13], [14]. This in turn negatively regulates the pro-survival PI3K/Akt signaling pathway. The phosphatidylinositol 3-kinase (PI3K) pathway regulates various cellular processes, including proliferation, growth, apoptosis and cytoskeletal rearrangement. PI3Ks are heterodimeric lipid kinases that are composed of regulatory and catalytic subunits encoded by different genes [15]. One of the main functions of PI3K is to synthesize the second messenger PtdIns(3,4,5)P3 (PIP3) from PtdIns(4,5)P2 (PIP2). AKT, a serine/threonine kinase that has a wide range of substrates, is activated by recruitment to the plasma membrane through direct contact of its pleckstrin-homology (PH) domain with PIP3, and phosphorylation at Thr308 and Ser473. AKT acts downstream of PI3K to regulate many biological processes, such as proliferation, apoptosis and growth, but other signaling pathways are also known to be regulated by PI3K activity and might be involved in PI3K-mediated tumorigenesis. PTEN is one of the most commonly lost tumor suppressor genes in human cancer. During tumor development, mutations and deletions of PTEN occur that inactivate its enzymatic activity leading to increased cell proliferation and reduced cell death. Frequent genetic inactivation of PTEN occurs in glioblastoma, endometrial cancer, prostate cancers; and reduced expression is found in many other tumors such as lung and breast cancer [15]. In this study Western analysis also showed a decrease in IL-32 expression after the inhibition of miR-23b-3p in A-498 cells. We did not monitor IL-32 in the culture media. IL-32 is a cytokine and it’s pro-cancerous role has been documented in several studies [16], [17], [18]. The PI3-kinase pathway has been shown to induce IL-32 expression in a number of studies [19], [20], [21], [22], especially in pancreatic cancer where its pro- cancerous role has been established. Therefore, these studies suggest that the reason for the decreased IL-32 expression upon knock down of miR-23b-3p is that it also affects the expression of PI3-kinase and Akt. A software search for miR-23b-3p binding sites on PI3-kinase and Akt was carried on however we did not find any evidence that PI3-kinase and Akt are direct targets of miR-23b-3p.

Genistein (40,5,7-trihydroxyflavone), one of the principal soy isoflavones, has a wide array of chemopreventive actions. The anticancer effects of genistein have been ascribed to several signaling pathways and mechanisms that affect cell cycle arrest, apoptosis, invasion, metastasis and angiogenesis, attributes that could potentially prevent tumor initiation and progression [23], [24]. Genistein is abundant in soy products and has been identified as an inhibitor of protein tyrosine kinases and thus may play a key role in cell growth and apoptosis [25], [26]. It has been reported to have estrogenic properties and anti-neoplastic activity in multiple tumor types [27]. In a previous study Hillman et. al. have shown that the combination of genistein with primary tumor irradiation acts effectively as a therapeutic approach, in established kidney tumors, due to tumor growth being inhibited at both the primary and metastatic sites. In their study genistein alone had a tendency to stimulate growth of primary kidney tumor and increase metastasis [28]. Thus these findings prompted us to monitor the expression of miRNA-23b-3p after treatment of A-498 cells with different concentrations of genistein. We found a 50% decrease in the expression of miR-23b-3p after treatment of A-498 cells with 25 µM genistein for 96 hours (Figure 8). In a previous study [29] we also reported a decrease of miR-21 expression in mouse xenograft tumors formed after injecting 25 µM genistein treated A-498 cells subcutaneously in nude mice as compared to smaller tumors formed by untreated A-498 cells. We also measured the expression of miR-23b-3p in those tumors and could not find any significant change in its expression.

In summary, miR-23b-3p is an oncogene in RCC and directly inhibits the PTEN tumor suppressor gene. Thus miR-23b-3p may be useful as a renal cancer diagnostic marker and as a therapeutic target for the treatment of renal cell carcinoma.

## Materials and Methods

### Ethics Statement

Formalin-fixed, paraffin-embedded (FFPE) renal cancer samples were obtained from the San Francisco Veterans Affairs (VA) Medical Center. Written informed consent was obtained from all patients and the study was approved by the UCSF Committee on Human Research (Approval number: H9058-35751-01).

### Cell Lines and Cell Culture

Human renal cancer cell lines A-498, ACHN, Caki-1, Caki-2, and a normal renal cell line (HK-2) were obtained from the American Type Culture Collection (ATCC, Manassas, VA, USA). Integrity of the cell lines was confirmed by the ATCC using DNA (STR) profiling. Normal renal HK-2 cells were cultured as a monolayer in Keratinocyte Serum Free Medium (K-SFM) supplemented with 0.05 mg/ml bovine pituitary extract (BPE), 5 ng/ml human recombinant epidermal growth factor (EGF) (Life Technologies/Invitrogen, Carlsbad, CA, USA), 10% fetal bovine serum (Atlanta Biologicals, Lawrenceville, GA, USA), 50 mg/ml penicillin and 50 mg/ml streptomycin (Invitrogen, Carlsbad, CA, USA). The renal cancer cell lines A-498 and ACHN were cultured as a monolayer in Eagle’s Minimum Essential Medium, (UCSF Cell Culture Facility, San Francisco, CA, USA). Caki-1 and Caki-2 were cultured the same way in McCoy’s5A media (UCSF Cell Culture Facility, San Francisco, CA, USA). All cell lines were maintained in an incubator with a humidified atmosphere of 95% air and 5% CO2 at 37°C.

### Genistein Treatment of A-498 Cells

A-498 cells (60–70% confluent) were treated with genistein (25 µM and 50 µM ). Genistein (Sigma-Aldrich Corp., St Louis, MO, USA) was dissolved in DMSO, and cells treated with vehicle (DMSO) served as control. Fresh genistein was administered everyday with a change of medium, and the cells incubated for 4 days.

### Quantitative Real-time PCR

For real-time polymerase chain reaction (PCR), complementary DNA was amplified with Inventoried Gene Assay Products containing two gene-specific primers and one TaqMan MGB probe (6-FAM dye-labeled) using a TaqMan Universal Fast PCR Master Mix in a 7500 Fast Real-Time PCR System (Applied Biosystems, Foster City, CA, USA). Thermal cycling conditions included 95°C for 20 seconds(s), 40 cycles of 95°C for 3s and 60°C for 30s according to the TaqMan Fast Universal PCR protocol. Total microRNA was extracted using a miRNeasy kit from Qiagen (Valencia, CA, USA). For miRNA real-time experiments the cDNA strand was synthesized using an Applied Biosystems Taqman MicroRNA Reverse Transcription kit (Applied Biosystems, Foster City, CA, USA), with 200 ng of total extracted miRNA. RNU48 was used as an endogenous control. It was also used as an endogenous control for real-time PCR experiments, where miRNAs were isolated from formalin-fixed paraffin-embedded (FFPE) renal samples. Total miRNA was extracted from FFPE samples using a miRNA FFPE kit from Qiagen.

### Micro Dissection of Renal Cancer Tissues and RNA Extraction from FFPE Human Renal Tumor Samples

Adjacent normal and cancerous renal tissues were obtained from 29 representative FFPE tissue blocks of radical nephrectomy specimens from the Pathology Department of the Veterans Affairs (VA) Medical Center of San Francisco. The blocks were from kidney cancer patients who were operated on at the VA Medical Center between 1980–2009. Sections (4 µm) of the blocks were prepared, H&E stained, and slides were reviewed by a board certified pathologist to mark the normal and cancer areas. Subsequently, 12 µm slides were made from the blocks and micro dissection was performed using the marked H&E stained slides as a template. microRNA extraction was done using a Qiagen FFPE miRNA extraction kit. The levels of miR-23b-3p were assessed by the Taqman miR assay as described above. Following PCR, relative miR-23b-3p expression levels in cancerous regions were normalised to their adjacent normal counterparts.

### Cell Transfection

A498 and Caki-2 cells were transiently transfected with either miRNA-23b-3p inhibitor (*mir*Vana® miRNA inhibitor Applied Biosystems, Assay ID no. MH10711) or anti-miR negative control #1 (from Ambion, Austin, TX, USA Catalog no. AM17010), using X-tremeGENE siRNA transfection reagent (Roche Diagnostics Indianapolis, IN, USA) according to the manufacturer’s protocol. In brief, cells were seeded in 6 well plates (Nunc, Roskilde, Denmark) 24 h before transfection and transiently transfected at a confluency of 40–50%. Mock transfection, with the transfection reagent, was also used as a control. The transfection mixture was dissolved in Opti-MEM serum-free media (UCSF Cell Culture Facility, San Francisco, CA, USA) and at the time of transfection cells were seeded in Eagle’s Minimum Essential Medium (UCSF Cell culture facility), with 10% FBS (Atlanta Biologicals, Lawrenceville, GA, USA) and no antibiotics. On the following day the media was changed to Eagle’s Minimum Essential Medium containing both FBS and 1% antibiotic (penicillin–streptomycin, 100x, UCSF Cell Culture Facility). Cells were pelleted after 72 h of transfection for flow cytometry, RNA and protein extraction.

### Cell Cycle Analysis

Cell cycle analysis was performed 72 h after transfection. The cells were harvested, washed with cold PBS, (UCSF Cell Culture Facility), and resuspended in the nuclear stain 4′,6-diamidino-2-phenylindole (Beckman Coulter, Brea, CA, USA). Stained cells were immediately analysed with a flow cytometer (Cell Lab Quanta SC; Beckman Coulter).

### Apoptosis Assay

For apoptosis, cells at 72 h after transfection were dual stained with the viability dye 7-amino-actinomycin D(7-AAD) and Annexin V-FITC using an Annexin V-FITC/7-amino-actinomycin D kit (Beckman Coulter) according to the manufacturer’s protocol. Stained cells were immediately analysed by flow cytometry (Cell Lab Quanta SC; Beckman Coulter).

### Migration and Invasion Assays

A cytoselect 24-well cell migration and invasion assay kit (Cell Biolabs, Inc., San Diego, CA) was used for migration and invasion assays at 72 hrs after transfection (8 µm, Colorimetric format) according to the manufacturer’s protocol.

### Western Analysis

Whole-cell extracts were prepared in radioimmunoprecipitation assay buffer (RIPA; Thermo Scientific, Rockford, IL, USA; 50 mmol l–1 Tris (pH 8.0), 150 mmol l–1 NaCl, 0.5% deoxycholate, 0.1% SDS and 1.0% NP-40) containing a protease inhibitor cocktail (Roche, Basel, Switzerland). Protein assays were performed using a BCA Protein assay kit (Pierce/Thermo Scientific, Rockford, IL, USA) according to the manufacturer’s instructions. Total protein (40 µg) was electrophoresed in 12% SDS–PAGE gels, and Western blotting was carried out using standard protocols and proteins detected by chemiluminescence. Antibodies, including PTEN (cat. no. 9552S) and Akt (cat. no. 4691S) were purchased from Cell Signaling (Danvers, MA, USA), PI3-kinase (cat no. ab69870) and IL-32 (cat no. ab62580) were from Abcam (Cambridge, MA, USA), whereas GAPDH (cat. no. sc-32233) was from Santa Cruz Biotechnology (Santa Cruz, CA, USA).

The protein expression levels were quantified by optical densitometry using ImageJ Software version 1.36b (http://rsb.info.nih.gov/ij/). Fold change was calculated as the ratio between the net intensity of each sample transfected with anti-miR-23b-3p divided by GAPDH and the negative control transfected samples divided by the GAPDH (Anti-miR-23b-3p transfected samples/GAPDH)/(Neg Control miR transfected samples/GAPDH).

### Luciferase Reporter Assay

Cells in 24-well plates were transfected with 30 nM pre-miR negative control (NC) or pre-miR-23b-3p (Applied Biosystems) and PTEN construct (Catalog no. HmiT015535, Genecopoeia, Rockville, MD, USA) or control construct, pEZX-MT01 (Genecopoeia) using Lipofectamine 2000 (Invitrogen), according to the manufacturer’s instructions. The control construct lacked miR-23b-3p target sites. All transfection experiments were performed in triplicate. Luciferase activity was assayed at 48 h after transfection, using a dual-luciferase reporter assay system (Promega).

### Immunohistochemistry

Immunostaining was done on renal cancer tissue slides [made from formalin-fixed, paraffin-embedded (FFPE) renal cell cancer tissue blocks using a microtome (Leica)]. The slides were deparaffinized and antigen retrieval was carried out by microwaving the slides in 10 mmol/L sodium citrate buffer. Slides were incubated overnight with anti-PTEN antibody (Cell signalling). The staining was done using the LABVISION Corporation (Thermo Fisher Scientific) Anti-Rabbit Staining System (LOT-RHD 80924) as per manufacturer’s instructions.

### Statistical Analysis

Statistical analysis was performed using StatView version 5.0 for Windows. Student’s t-test was used to compare the different groups. p-values of <0.05 were regarded as statistically significant. Chi-square test was performed to determine the correlation between miR-23b-3p expression levels and PTEN protein expression levels in tissue samples.
